# A Novel Process for the Containment of SO_2_ Emissions from Class C Fly Ash in the Fired Materials by Haüyne Formation

**DOI:** 10.3390/ma15196701

**Published:** 2022-09-27

**Authors:** Radomir Sokolar, Martin Nguyen

**Affiliations:** Faculty of Civil Engineering, Institute of Technology of Building Materials and Components, Brno University of Technology, Veveri 331/95, 602 00 Brno, Czech Republic

**Keywords:** fly ash, ceramic body, firing, sulfur dioxide emissions, haüyne

## Abstract

Class C fly ash has been receiving increasing attention due to the gradual transition of thermal power plants all over the world to the fluidized bed combustion technology with sulfur dioxide emissions capture. This research investigates the utilization of class C fly ash in fired ceramic materials with simultaneous efficient and novel containment of sulfur dioxide emissions in the flue gas during firing. A number of experiments were conducted by addition of sodium water glass with different molar ratios of SiO_2_:Na_2_O, sodium carbonate, and different ratios of sodium carbonate to water glass to the class C fly ash to examine the optimal combination and quantity for the creation and formation of the mineral phase haüyne which resulted in reduction and containment of SO_2_ emissions. Results revealed that a 12% dose of sodium water glass with a low molar ratio of 1.7 (SiO_2_:Na_2_O) combined with class C fly ash was more effective in the formation of haüyne and the resulting decrease of SO_2_ in the flue gas was more substantial. The newly formed mineral phase haüyne was identified by an X-ray diffraction analysis and scanning electron microscopy with energy dispersive X-ray spectroscopy. Outcomes reveal a potential for utilization of class C fly ash in the fired materials by containment of sulfur dioxide into their structure.

## 1. Introduction

Class C fly ash (CCFA), also known as fluidized fly ash, is an energy by-product of the process of combustion of milled coal-limestone (dolomite) mixture in the fluidized bed reactors within thermal power plants. Fluidized bed technology is a modern method employed for the limitation of harmful emissions into the air (particularly sulfur dioxide). The fluidized bed reactors usually use low temperatures combustion (around 800 °C). In contrast, the traditional combustion of pure coal is usually at about 1400 °C (fly ash forms at higher temperatures). The total worldwide production of fly ash is in the order of hundreds of millions of tons each year, where the majority is being deposited in landfills. However, many countries have begun to use fly ash in ever-increasing quantities to meet the goals for sustainable development and reduction of environmental pollution. The production of both fly ashes (Class C and F) in the Czech Republic alone comprises about 11 million tons per year—9.4 million tons of Class F and 1.6 million tons of Class C fly ash. CCFAs are fly ashes derived from the combustion of the milled coal-limestone mixture in fluid beds. Standard ASTM C618-12a divides fly ashes into two groups: Class F—classic high-temperature fly ash and Class C—fluidized fly ash (Table 1).

There are numerous studies published on the use of different kinds of fly ashes in nonfired materials—concrete technology, geopolymers [1,2], solidification of dangerous substances, or the stabilization of unstressed layers for roads [3]. The available research is limited to the Class F fly ash, as publications about the utilization of CCFA in ceramic technology are rare. However, Erol et al. [4] investigated the possible utilization of CCFA in glass production, and Fernández-Pereira et al. [5] studied the utilization of fly ash derived from fluidized reactors for brick manufacturing. Although their works dealt with the utilization of CCFA, these studies did not address the extent of sulfur dioxide emissions in flue gas during firing. The mixture of Class F fly ash and kaolinic stoneware clay for the production of single-fired dry-pressed ceramic tiles was used [6,7,8]. The fired bodies indicate a higher shrinkage after the firing in comparison with the standard bodies produced from natural raw materials. The addition of limestone to the (Class F) fly ash-clay mixture decreases the firing shrinkage and bending strength of the fired bodies [9,10]. The fundamental component in the raw materials mixture for the preparation of ceramic tiles is alkali-activated coal Class F fly ash (e.g., quartz, hematite, mullite, glass phase) [11,12]. CCFA has been successfully used for the preparation of glass-ceramic materials [13,14,15,16] and fired bricks [17,18,19]. The milling of fly ashes improves the sintering activity of fly ash-clay mixtures [6,20]. The use of different CCFAs with a CaO content of 6.76–37.80% obtained from thermal power plants has been studied for the production of glass materials [4]. Research indicates that increasing the quantity of CCFA in the raw materials mixture of clay and class C fly ash directly increases the amount of SO_2_ in the flue gas during firing [21]. In general, previously published results describe the utilization of CCFA in raw materials mixtures for ceramic technology. However, these publications do not address the sulfur dioxide emissions during firing due to anhydrite decomposition. Evidence indicates that the practical utilization of CCFA in real-fired ceramic raw materials requires solving the dissolution of anhydrite CaSO_4_ and SO_2_ emissions during firing. The creation of anhydrite in CCFAs during the combustion of the milled coal and limestone mixture inside the fluidized boiler can be described by chemical equations in two phases [22]:

1. Decomposition of limestone:

CaCO_3_ → CaO + CO_2_. (1)

2. Formation of anhydrite:

2CaO + 2SO_2_ + O_2_ → 2CaSO_4_. (2)

The decomposition of CaSO_4_ during the firing of dry-pressed bodies based on pure CCFA intensively produces calcium oxide (CaO), sulfur dioxide (SO_2_) gas, and oxygen (O_2_)—reverse reaction of Equation (2). As a result of the decomposition of pyrite FeS_2_ (per the exothermic reaction in Equation (3)), an increased amount of SO_2_ can be expected in the flue gas within the temperature range of 350–550 °C [23].

4FeS_2_ + 11O_2_ → 2Fe_2_O_3_ + 8SO_2_. (3)

The reverse chemical reaction of Equation (2)—anhydrite decomposition—is realized during the firing of dry-pressed bodies, based on the pure unmilled CCFA at the soaking time, at 1200 °C. When the milled CCFA was used, the decomposition of anhydrite started at lower temperatures (about 1040 °C). Sulfur dioxide appears in the flue gases from low temperatures close to 940 °C (milled fly ashes) or 990 °C (unmilled fly ashes) due to the decomposition of anhydrite CaSO_4_ in dry-pressed clay-fly ash bodies [24,25]. Haüyne Na_4_Ca(Si_3_Al_3_)O_12_(SO_4_) = 2Na_2_O·CaO·3SiO_2_·Al_2_O_3_(SO_4_) [26] is a tectosilicate sulfate mineral from the sodalite group (feldspathoids). Haüyne forms a solid solution with nosean Na_8_Al_6_Si_6_O_24_(SO_4_).H_2_O, and sodalite Na_8_(Al_6_Si_6_O_24_)Cl_2_. Nosean with sodalites are isomorphic. Sodalites can be converted into nosean by heating them in molten sodium sulfate. Furthermore, haüyne can be converted to sodalite by heating it within a NaCl solution [23]. Haüyne and nosean are easily identifiable through X-ray diffraction [27,28,29] with isometric-hextetrahedral structures and approximately the same interplanar spacing d (2θ) (intensity) for haüyne, which is 3.72 (23.90°) (100%), 2.63 (34.06°) (50%), 6.45 (13.72°) (30%) while the intensity values for nosean are 3.71 (23.97°) (100%), 2.628 (34.09°) (75%), 6.45 (13.72°) (70%), respectively. Table 2 presents the conventional chemical composition of natural minerals of haüyne and nosean.

Despite abundant laboratory research [1,2,3,4,5,6,7,8,9,10,11,12,13,14,15,16,17,18,19,20,21], there are no examples of a broad application of CCFA within the production of fired building materials because the decomposition of anhydrite and the resulting re-release of sulfur dioxide (SO_2_) into the atmosphere has not been solved. The previous research only provides information about the temperature ranges of the SO_2_ content in the flue gas during the firing of pure CCFA bodies or the mixtures containing CCFA [24,25]. The finding of new ways for the utilization of CCFA is important for the environment and sustainable development since the CCFA is produced in very large quantities all over the world, and this number is predicted to continue to grow due to the transition from conventional high-temperature coal burning to fluidized bed reactor coal burning due to the efforts to reduce emissions released into the air. In this research, the possible utilization of CCFA in ceramic technology is investigated with simultaneous measurement of the SO_2_ content in flue gas during the firing of dry-pressed bodies based on typical CCFA through the addition of various sodium oxide sources for the possible containment of sulfur in the fired bodies in the form of crystallization of new mineralogical phase containing sulfur oxide in its structure.

## 2. Materials and Methods

CCFA (thermal power plant, Hodonín, Czech Republic), denoted as CCFA-H, was used as a raw material for the production of test samples. CCFA-H is characterized by its high SO_3_ content in the form of mineral anhydrite (CaSO_4_), calcium oxide (CaO) (up to 15%), calcite (CaCO_3_), and amorphous aluminosilicate phase. The chemical composition (ICP 05: EN ISO 11885) of CCFA-H (Table 3) is typical for Class C fly ashes.

**Figure 1:**
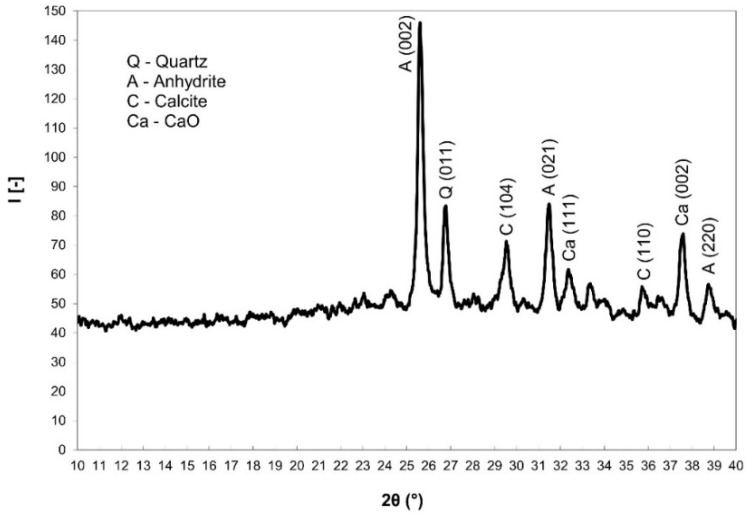
Mineralogical composition of the used CCFA (CCFA-H) via XRD patterns.

Figure 1 contains the mineralogical composition of the used CCFA according to the X-ray diffraction (XRD) patterns, identified minerals were quartz SiO_2_ (PDF card 96-500-0036), anhydrite CaSO_4_ (PDF card 96-900-4097), calcite CaCO_3_ (PDF card 96-900-0967), lime CaO (PDF card 96-900-6712), and hematite Fe_2_O_3_ (PDF card 96-900-9783). The background curvature also indicated the presence of an amorphous aluminosilicate phase. Rietveld quantitative analysis—28.0% anhydrite CaSO_4_, 18.6% quartz SiO_2_, 30.5% calcite CaCO_3,_ 6.7% hematite Fe_2_O_3_, 2.0% CaO, and 14.2% amorphous aluminosilicate phase are the main phases. Table 4 contains the crystallographic parameters of identified minerals.

**Figure 2:**
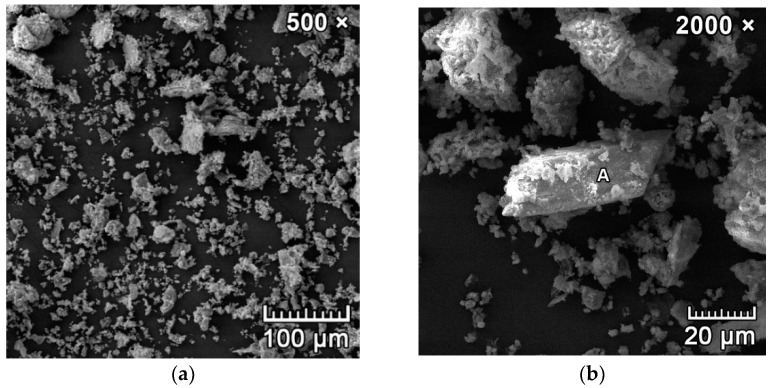
SEM microphotographs of used CCFA; (**a**) overall microstructure of particles; (**b**) detail of anhydrite crystal (marked as A) surrounded by clusters of smaller quartz, calcite, and amorphous particles.

Figure 2a,b represents the microstructure of the used CCFA. The anhydrite crystal can be seen in Figure 2b marked with letter A, the other fine particles were located in clusters and contained quartz, hematite, calcite, and amorphous aluminosilicate particles.

The granulometry of CCFA-H according to the residue on a screen (63 µm size) was determined, and the resulting 28.6% is conventional for this type of fly ash. Used CCFA was not treated (milled) for the experiment. However, according to [6], the granulometry of CCFAs influenced the temperature of the SO_2_ content in the flue-gas start.

The sources of Na_2_O for the preparation of test samples included industrially manufactured sodium water glasses from a Czech producer (Vodni Sklo, a.s.) with molar ratios of 1.7 and 3.5, respectively, and commercially sold dry sodium carbonate (Na_2_CO_3_).

The experiments were conducted in three consecutive stages. In the first stage, the assessment of the ability of sodium water glass to reduce the SO_2_ content in the flue gas during the firing of dry-pressed bodies based on CCFA via haüyne (nosean) crystallization was studied. Furthermore, the optimal quantity and molar ratio of the sodium water glass was derived. In the second stage, the assessment of the theory of using sodium carbonate or the mixture of sodium water glass with sodium carbonate-CCFA for haüyne creation of the CCFA-H body and the resulting decrease in SO_2_ content of the flue gas during the firing was studied. The third stage dealt with the identification of the presence of haüyne in a fired body via X-ray diffraction analysis, scanning electron microscopy (SEM) with an energy-dispersive X-ray spectroscopy (EDX) analysis, and sulfur content (chemical analysis) in the fired CCFA bodies was carried out.

The preparation of the test samples was carried out in subsequent steps from the untreated (unmilled) CCFA-H and different sources of Na_2_O (i.e., water, water glass, or sodium carbonate). First, the CCFA-H was moistened with water to achieve 18% humidity while the additives (i.e., sodium water glass or sodium carbonate) were incorporated into the water. All samples were prepared with similar contents of the liquid phase (water and sodium water glass). Table 4 and Table 5 provide details of the specific composition of the individual mixtures and their designations. Then, the moistened mixtures were pressed through the 1 mm sieve to prepare granulates, which were then homogenized for 24 h in the laboratory rotary homogenizer to reach the homogenous moisture of granulate.

Afterwards, the samples were uniaxially pressed at a pressure of 20 MPa and dried at 110 °C, the dimensions of the test samples were 100 × 50 × 10 mm, and six samples for each raw materials mixture were prepared. Dried green bodies were fired in an electric laboratory muffle furnace (5-liter volume) at temperatures of 1150–1200 °C to detect the SO_2_ content in the flue gas with a heating rate of 10 °C/min and 10 min of soaking time at the maximum temperature, respectively. The Testo SE 340 flue-gas analyzer (calibrated by the producer) consistently calculated the sulfur dioxide content in the flue gas during the firing process whenever a sample (50 × 50 × 10 mm) was fired.

For determination of the mineralogical composition, the XRD analysis (PANalytical Empyrean, PANalytical B.V., Almelo, The Netherlands) was used, which incorporated the CuKα as a radiation source with an accelerating voltage of 45 kV and a beam current of 40 mA, a diffraction angle 2θ in the range from 10° to 40° with a step scan of 0.01°. Rietveld refinement was carried out to determine the amount of haüyne in the sample. Quantitative analysis was performed using fluorite (CaF_2_) as an internal standard (15 wt.% per sample). SEM method (Tescan Mira3, Tescan Orsay Holding a.s., Brno, Czech Republic) with an integrated EDX probe was used for the identification of haüyne crystals in the fired bodies. 

## 3. Results of Experiments

### 3.1. Phase 1: Calculation of the Optimal Quantity and Molar Ratio of the Sodium Water Glass

The test samples made from pure CCFA-H and water/sodium water glass with different molar ratios (1.7–3.5) were prepared for this phase of research (Table 5). 

**Figure 3:**
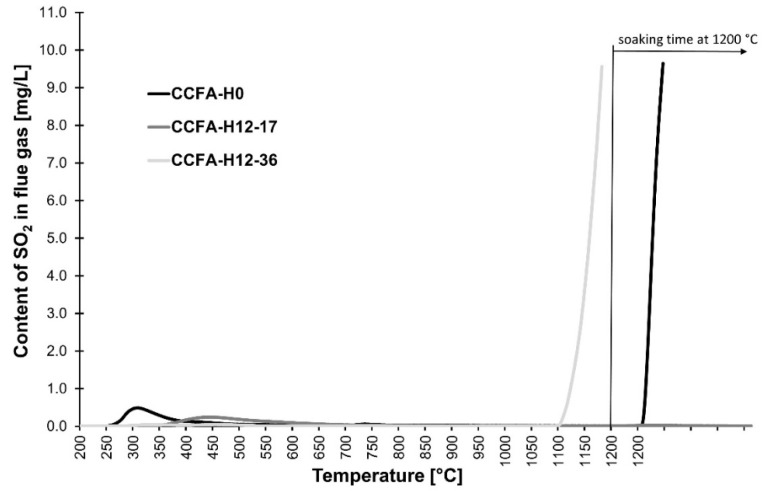
The content of SO_2_ in the flue gas during the firing of test samples CCFA-H0, CCFA-H12-17, and CCFA-H12-36 depending on the firing temperature.

The sulfur dioxide was released from all the samples fired except for the CCFA-H12-17 sample, which was due to the decomposition of the anhydrite contained in the CCFA-H. The admixture of water glass generally reduced the temperature of the initial SO_2_ content in the flue gas during the firing process (Figure 3). 

**Figure 4:**
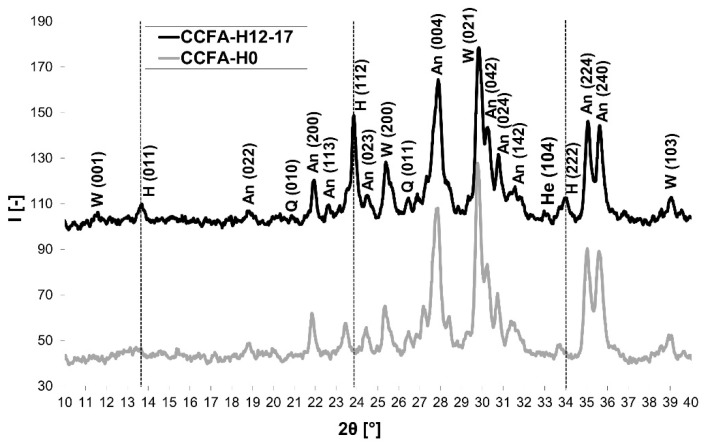
The comparison of the mineralogical composition (XRD patterns) of fired bodies: CCFA-H0 vs. CCFA-H12-17 with no sulfur dioxide content in the flue gas during firing (W: Wollastonite, H: Haüyne, An: Anorthite, A: Anhydrite, Q: Quartz, He: Hematite).

Elimination of the sulfur dioxide content in the flue gases occurred when a 12% content of sodium water glass (in the raw materials mixture) with a low molar ratio (1.7) was used. This result can be attributed to the binding of sulfur to the structure of the emerging mineral haüyne, as demonstrated by the XRD analysis (Figure 4). Identified minerals were haüyne Na_4_Ca_2_Al_6_Si_6_O_22_S_2_(SO_4_) (PDF card 96-101-1245), anorthite CaAl_2_Si_2_O_8_ (PDF card 96-900-0362), wollastonite CaSiO_3_ (PDF card 96-900-5778), quartz SiO_2_ (PDF card 96-500-0036), and anhydrite CaSO_4_ (PDF card 96-900-4097). Samples fired at 1200 °C showed a slight increase in emissions in the temperature range of 250–550 °C (pyrite decomposition [23]). Table 6 contains the crystallographic parameters of identified minerals.

**Figure 5:**
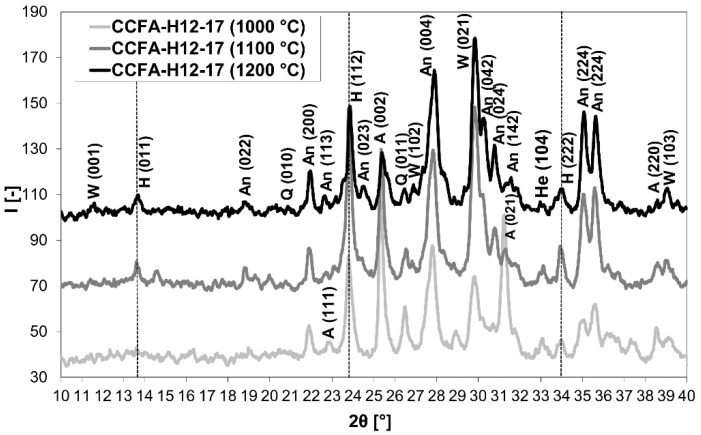
The effect of the firing temperature on the mineralogical composition of the CCFA-H12-17 body (W: Wollastonite, H: Haüyne, An: Anorthite, A: Anhydrite, Q: Quartz, He: Hematite).

The formation of the mineral haüyne in the CCFA-H12-17 sample after the firing at 1000 °C was comparable to higher temperatures, while the reduction of the anhydrite content was dependent on the rising firing temperature (Figure 5)—the content of haüyne was 14.2% (1000 °C), 19.8% (1100 °C), and 18.7% (1200 °C) in the fired bodies depending on firing temperature. Therefore, the mineralogical composition of the fired body is related to that of the anorthite, wollastonite, hematite, and quartz.

This phase aimed to verify the binding of the sulfur dioxide during the firing of CCFA dry-pressed bodies into the emerging mineral haüyne. The combination of a sufficient amount of sodium oxide, in the form of sodium water glass (12%), with a low molar ratio (1.7) is fundamental for the formation of haüyne.

### 3.2. Phase 2: The Determination of the Soda/Soda-Water Glass Mixture Addition for the Reduction of SO_2_ Emissions

In the experiments that followed, soda (sodium bicarbonate) with a weight of 7% and 9% (source of sodium ions) and a mixture of water glass and soda with a 10% weight were used, respectively (Table 7). The firing temperature was 1150 °C, which corresponds to the actual firing temperature of ceramic tiles [23], indicating that the research can be realistically applied in the future.

**Figure 6:**
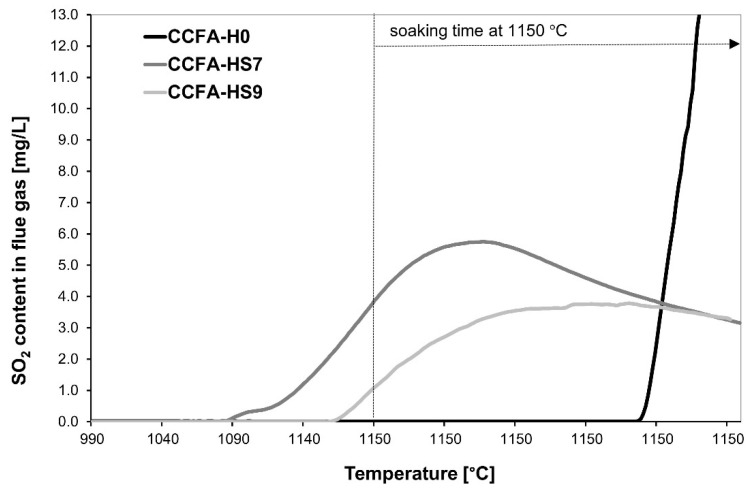
The sulfur dioxide content in the flue gas during the firing process is dependent on the addition of sodium carbonate.

**Figure 7:**
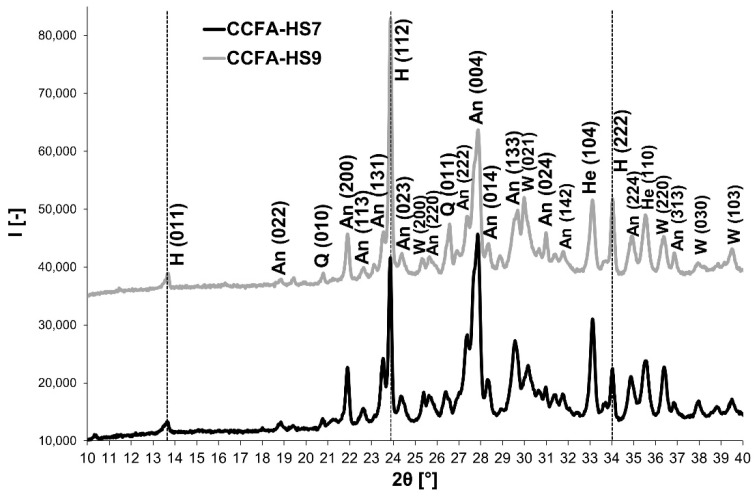
XRD patterns: The mineralogical composition of CCFA-H fired bodies depending on the different contents of sodium carbonate (W: Wollastonite, H: Haüyne, An: Anorthite, Q: Quartz, He: Hematite).

It is evident from Figure 6 that the addition of sodium carbonate as a source of Na_2_O has similar effects to the sodium water glass—test samples containing sodium carbonate indicate a significant decrease in the SO_2_ content in the flue gases (Figure 6) because of the mineral haüyne formation, which was identified in the fired bodies in both sodium carbonate doses (7% and 9%) (Figure 7). 

The addition of sodium carbonate causes SO_2_ to escape from the dry-pressed CCFA body at lower temperatures than a body without its admixture (CCFA-H0). All samples containing the Na_2_O source revealed a significant reduction of sulfur dioxide in the flue gas (Table 8). The reduction of SO_2_ emissions compared to the sample without the source of Na_2_O was decreased by 34.4–82.9%. The highest reduction of SO_2_ emissions occurred in sample CCFA-HB (marked in bold), with an 82.9% decrease.

The XRD-analysis diffractograms (Figure 7) indicate that in both samples containing sodium carbonate, after firing at 1150°C, the haüyne is formed, followed by the anorthite, the wollastonite, and a small amount of quartz. A sample with a higher sodium carbonate content (CCFA-HS9) reveals that a higher amount of haüyne is formed at the expense of the anorthite.

**Figure 8:**
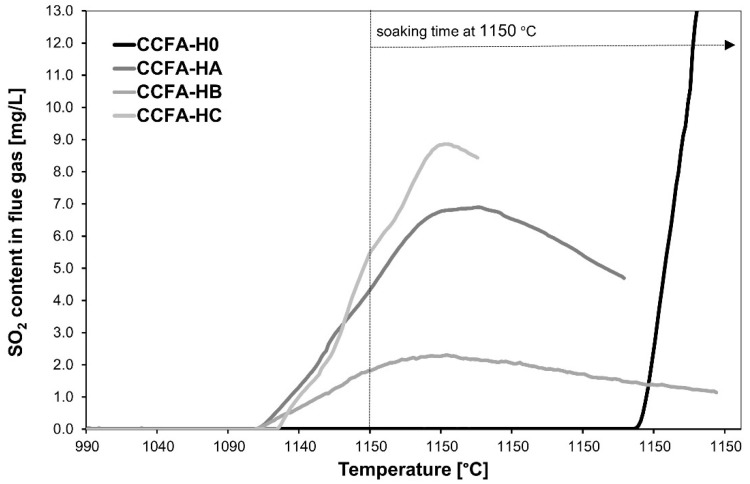
The content of SO_2_ in the flue gas during the firing of CCFA dry-pressed bodies—the effect of different ratios of sodium carbonate and sodium water glass.

The combination of both tested sources of Na_2_O—sodium carbonate and sodium water glass with a molar ratio of 1.7—appears to be very effective in eliminating the content of sulfur dioxide in the flue gas during firing (Figure 8). 

**Figure 9:**
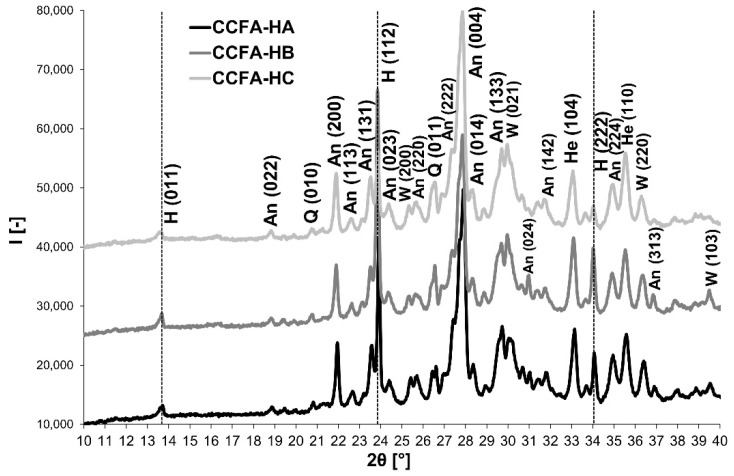
XRD patterns of CCFA-H (A, B, C) fired bodies with different ratios of Na_2_O sources - sodium carbonate and sodium water glass, according to Table 7 (W: Wollastonite, H: Haüyne, An: Anorthite, Q: Quartz, He: Hematite).

The tested combinations (Table 7) of ratios of sodium carbonate (S) and sodium water glass with a molar ratio of 1.7 (WG) are 2:1, with the lowest SO_2_ content in the flue gas and the highest content of haüyne mineral in the fired body at 1150 °C (Figure 7). It is possible to find a connection between the content of SO_2_ in the flue gas (Figure 8) and the content of the mineral haüyne in the body (Figure 9). The lowest content of SO_2_ in flue gases (CCFA-HB) corresponds to the highest content of the mineral haüyne in bodies (HA: 14.7%, HB: 18.9%, HC: 13.1%, calculated according to Rietveld analysis). The results of the XRD analysis correspond to the results of the flue-gas analysis (Figure 8). Therefore, the higher the amount of sodium carbonate in the sample, the more haüyne is present, and the lower the anorthite.

### 3.3. Phase 3: Identification of the Presence of Haüyne in the Microstructure of the Fired Body

**Figure 10:**
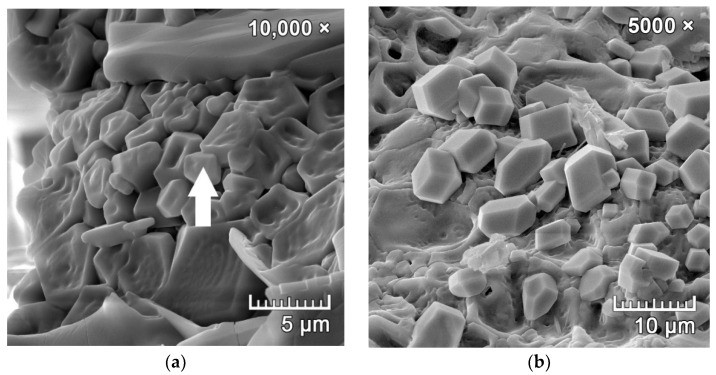
The microstructure of a fired CCFA-H12-17 body—(**a**,**b**) the identification of the haüyne crystals and location of the EDX probe (arrow).

A CCFA-H12-17 sample fired at 1200 °C (the haüyne was identified by XRD analysis—Figure 4) was selected for the analysis of the microstructure by SEM. The microstructure of the sample and sought out a crystal for the investigation (Figure 10a,b) were monitored. A secondary electron (SE) detector scanned the samples using an EDX element probe to verify the presence of the mineral.

**Figure 11:**
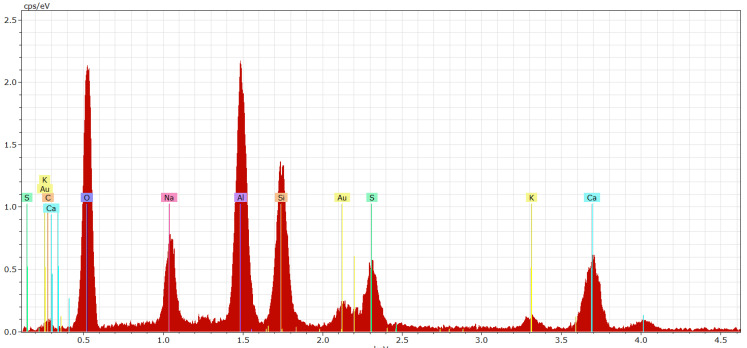
The result of the EDX analysis of the haüyne crystal (placed according to Figure 10a).

The result of the EDX analysis (Figure 11) indicates that the haüyne crystals identified through SEM (Figure 10a,b) contain sulfur (S), calcium (Ca), sodium (Na), silicon (Si), aluminum (Al), oxygen (O) and potassium (K), which are the atoms that make up the haüyne structure (Table 2).

## 4. Discussion

Haüyne appears in fired CCFA bodies containing 12% of sodium water glass from firing at 1000 °C (Figure 5), which is still below the temperature of anhydrite decomposition and the start of SO_2_ presence in flue gas (Figure 3). From this, it is clear that the crystallization mechanism of haüyne uses solid sulfur (anhydrite). 

The following schematic chemical reactions (4) and (5) are assumed during the firing of CCFA (contains anhydrite CaSO_4_ and Al-Si amorphous phase Al_2_O_3_.SiO_2_, similar to that of metakaolin from the decomposition process of clay minerals in coals [30]) dry-pressed bodies containing sodium water glass Na_2_O.SiO_2_ with a molar ratio of 1.7 (Equation (4)) or sodium carbonate Na_2_CO_3_ (Equation (5)) = formation of haüyne 2Na_2_O·CaO·3SiO_2_·Al_2_O_3_(SO_4_):

2Na_2_O·SiO_2_ + CaSO_4_ + Al_2_O_3_·SiO_2_ → 2Na_2_O·CaO·3SiO_2_·Al_2_O_3_(SO_4_), (4)

2Na_2_CO_3_ + CaSO_4_ + Al_2_O_3_·SiO_2_ + 2SiO_2_ → 2Na_2_O·CaO·3SiO_2_·Al_2_O_3_(SO_4_) + 2CO_2_. (5)

The ability of sodium carbonate and sodium water glass to bind the sulfur in the body in the form of a crystallized haüyne was demonstrated by the chemical analysis of the fired CCFA-H0 body, with the highest SO_2_ content in the flue gas, and the CCFA-H12-17 body, with the lowest sulfur dioxide content in the flue gas (Table 9). The reduction of SO_2_ emissions in sample CCFA-HB compared to the sample without the source of Na_2_O (CCFA-H0) was decreased by 82.9%. The SO_3_ content of 3.42 % in the CCFA-H12-17 body is equivalent to the SO_3_ content of the original ash CCFA-H (Table 3). Thus, the sulfur in this body is bound in the detected mineral haüyne.

## 5. Conclusions

This research paper has provided a solution for the utilization of class C fly ash as a secondary raw material for the possible production of porous ceramic wall tiles with a typical firing temperature of about 1130 °C as a substitution for traditional natural raw materials (limestone, dolomite), which are the primary sources of calcium oxide in the ceramic wall tiles. 

The sulfur dioxide is bound in class C fly ash as mineral anhydrite which decomposes in temperatures from about 900 °C during firing. In this paper, it was experimentally found that the amount of SO_2_ retroactively released into the atmosphere, compared to the sample without the source of Na_2_O, was decreased by 34.4–82.9%. The best results were achieved by a 12 wt.% dose of sodium water glass with a low molar ratio of 1.7 combined with class C fly ash. It was the most efficient in the formation of new mineralogical phase haüyne, and the resulting decrease of SO_2_ in the flue gas was 82.9% during firing.

Appropriate combinations of class C fly ash with Na_2_O sources (10 wt.% of sodium carbonate to sodium water glass mixture with a weight ratio of 2:1, respectively) can produce a fired body during the firing with an in-situ capture of sulfur dioxide from class C fly ash, which binds the present sulfur into the structure of haüyne, thus preventing the retroactive release of SO_2_ emissions into the atmosphere. Haüyne was identified in the fired bodies by X-ray diffraction analysis and scanning electron microscopy with energy dispersive X-ray spectroscopy.

## Figures and Tables

**Table 1:** ASTM C618-12a classification of fly ashes.

Fly Ash	Σ(SiO_2_ + Al_2_O_3_ + Fe_2_O_3_)	SO_3_ Content	Loss on Ignition
Class C	≥50%	≤5%	≤6%
Class F	≥70%	≤3%	≤6%

**Table 2:** Chemical composition of natural haüyne (Badakhshan, Afghanistan [25]) and nosean (Covao, Cabo Verde [25]).

Mineral	SiO_2_	Al_2_O_3_	CaO	Na_2_O	K_2_O	MnO	SO_3_
Haüyne	34.28	28.70	7.11	18.04	0.09	0.03	12.54
Nosean	34.95	24.41	4.40	19.01	0.33	0.00	8.11

**Table 3:** Chemical composition of the used CCFA-H.

Oxide	SiO_2_	Al_2_O_3_	Fe_2_O_3_	TiO_2_	CaO	MgO	MnO	K_2_O	Na_2_O	SO_3_	LOI ^1^
Content [%]	32.45	16.08	6.67	0.60	24.52	3.41	0.00	0.63	0.10	4.04	4.43

^1^ Loss on ignition.

**Table 4:** Crystallographic parameters of identified minerals in CCFA.

Mineral	Crystal System	Lattice Parameters [Å; °]						Cell Volume [Å^3^]
		a	b	c	α	β	γ	
**Quartz**	Hexagonal	4.912	4.912	5.404	90	90	120	112.92
**Anhydrite**	Orthorhombic	6.995	6.245	6.993	90	90	90	305.48
**Calcite**	Hexagonal	4.984	4.984	17.121	90	90	120	368.31
**Lime (CaO)**	Cubic	4.937	4.937	4.937	90	90	90	120.33
**Hematite**	Hexagonal	5.0288	5.0288	13.730	90	90	120	300.70

**Table 5:** The composition and indication of test samples for Phase 1 of the experiments.

	CCFA-H0	CCFA-H6-17 CCFA-H6-35	CCFA-H9-17 CCFA-H9-35	CCFA-H12-17 CCFA-H12-35
Water	18%	12%	9%	6%
Water glass (1.7–3.5)	0%	6%	9%	12%

**Table 6:** Crystallographic parameters of identified minerals in test samples.

Mineral	Crystal System	Lattice Parameters [Å; °]						Cell Volume [Å^3^]
		a	b	c	α	β	γ	
**Haüyne**	Cubic	9.100	9.100	9.100	90	90	90	753.57
**Anorthite**	Triclinic	8.194	12.897	12.931	86.00	81.04	88.85	1345.52
**Wollastonite**	Triclinic	7.065	7.320	7.926	103.43	95.22	90.06	396.94
**Quartz**	Hexagonal	4.912	4.912	5.404	90	90	120	112.92
**Anhydrite**	Orthorhombic	6.995	6.245	6.993	90	90	90	305.48
**Hematite**	Hexagonal	5.0288	5.0288	13.730	90	90	120	300.70

**Table 7:** The composition and indication of test samples for Phase 2 of the experiments.

Indication	Admixture	Content/Ratio
**CCFA-HS7**	Sodium carbonate	7%
**CCFA-HS9**		9%
**CCFA-HA**	Sodium carbonate: sodium water glass (1.7)	10% (1:1)
**CCFA-HB**		10% (2:1)
**CCFA-HC**		10% (1:2)

**Table 8:** The temperatures of anhydrite decomposition start (Ts) and the maximum measured quantity of SO_2_ in the flue gas during the firing (SO_2_-max).

Batch	Ts [°C]	SO_2-max_ [mg/L]
CCFA-H0	1150	>13.0
CCFA-HS7	1078	6.39
CCFA-HS9	1145	5.75
CCFA-HA	1107	6.90
CCFA-HB	1100	2.31
CCFA-HC	1125	8.86

**Table 9:** The chemical analysis of the fired bodies—sulfur content depending on water glass content in raw materials mixture (0% vs. 12%).

	CCFA-H0	CCFA-H12-17
Total sulfur content as SO_3_ [%]	1.89	3.42
Total sulfur content [%]	0.76	1.37

## Data Availability

The data presented in this research paper are available upon request from the corresponding author.
